# Nasal commensal *Staphylococcus epidermidis* counteracts influenza virus

**DOI:** 10.1038/srep27870

**Published:** 2016-06-16

**Authors:** Hui-Wen Chen, Pei-Feng Liu, Yu-Tsueng Liu, Sherwin Kuo, Xing-Quan Zhang, Robert T. Schooley, Holger Rohde, Richard L. Gallo, Chun-Ming Huang

**Affiliations:** 1 Department of Veterinary Medicine, National Taiwan University, Taipei, Taiwan; 2 Department of Dermatology, University of California, San Diego, CA, USA; 3 Division of Infectious Diseases, Department of Medicine, University of California, San Diego, CA, USA; 4 Moores Cancer Center, University of California, San Diego, CA, USA; 5 Institut für Medizinische Mikrobiologie, Virologie und Hygiene, Universitätsklinikum, Hamburg, Germany

## Abstract

Several microbes, including *Staphylococcus epidermidis* (*S. epidermidis*), a Gram-positive bacterium, live inside the human nasal cavity as commensals. The role of these nasal commensals in host innate immunity is largely unknown, although bacterial interference in the nasal microbiome may promote ecological competition between commensal bacteria and pathogenic species. We demonstrate here that *S. epidermidis* culture supernatants significantly suppressed the infectivity of various influenza viruses. Using high-performance liquid chromatography together with mass spectrometry, we identified a giant extracellular matrix-binding protein (Embp) as the major component involved in the anti-influenza effect of *S. epidermidis*. This anti-influenza activity was abrogated when Embp was mutated, confirming that Embp is essential for *S. epidermidis* activity against viral infection. We also showed that both *S. epidermidis* bacterial particles and Embp can directly bind to influenza virus. Furthermore, the injection of a recombinant Embp fragment containing a fibronectin-binding domain into embryonated eggs increased the survival rate of virus-infected chicken embryos. For an *in vivo* challenge study, prior Embp intranasal inoculation in chickens suppressed the viral titres and induced the expression of antiviral cytokines in the nasal tissues. These results suggest that *S. epidermidis* in the nasal cavity may serve as a defence mechanism against influenza virus infection.

Bacterial interference, or bacteriotherapy, in which commensal bacteria are used to prevent colonization of the host by pathogens, is a promising strategy for combating infections^1^,^2^,^3^,^4^,^5^,^6^,^7^. Bacterial interference has been utilized in the clinic for the treatment of recurrent staphylococcal infections and for the prevention of urinary tract infections^8^. In addition, therapeutic application of bacterial interference by active colonization using a “non-pathogenic” *Staphylococcus aureus* (*S. aureus*) strain, 502A, was successful in controlling outbreaks of *S. aureus* infections in the 1960s and in treating patients with recurrent furunculosis^9^,^10^,^11^. More intriguingly, previous studies have shown that introduction of live *Staphylococcus epidermidis* (*S. epidermidis*) into patients who were *S. aureus* carriers significantly reduced the *S. aureus* colonization^12^. These findings indicate that it is safe to inoculate *S. epidermidis* into human nasal cavities as an interfering agent. *S. epidermidis* comprises 90–100% of the staphylococci from the human nasal cavity^13^,^14^. *S. epidermidis* is also a commensal bacterium in turkeys and chickens^4^,^5^,^6^, and *S. epidermidis* isolates from turkeys have been used as interfering agents to help control *S. aureus* infections^5^,^6^,^7^. Field administration of interfering *S. epidermidis* into 200,000 turkeys in 21 flocks decreased the *S. aureus* colonization and increased the survival rate of *S. aureus*-infected turkeys^5^. In addition, the injection of interfering *S. epidermidis* into embryonated eggs was shown to lead to a reduction in lethality following influenza virus challenge (A_2_J 305)^15^.

Influenza is a highly contagious infection of the upper respiratory tract of humans. The upper respiratory tract is usually the initial site of invasion of influenza virus; thus, effective anti-viral defences at the site of infection should inhibit the systemic spread of the virus. Vaccines have historically been the mainstay of infection control. However, influenza vaccines must be updated annually due to seasonal antigenic drifts^16^. Neuraminidase inhibitors, especially oseltamivir (Tamiflu), are the primary treatment for various influenza viruses, although they show limited efficacy if administered late in disease progression, and widespread use is likely to result in the emergence of resistant viral strains^17^. Thus, there is a need for novel antiviral agents with a lower tendency to induce resistance.

Here, we demonstrate that *S. epidermidis* significantly suppresses the infectivity of various influenza viruses via a giant extracellular matrix-binding protein (Embp). This work provides a potential therapeutic strategy for influenza virus infection. In particular, the use of nasal commensal bacteria as antiviral agents offers three unique advantages. (1) Unlike antibiotics, commensal bacteria are indigenous to humans and may have a lower risk of causing serious complications or generating resistant viruses. (2) *S. epidermidis* bacteria are also native inhabitants of birds^5^,^6^,^7^,^18^,^19^ and pigs^19^,^20^ and have been used as interfering agents for the prevention of pathogen colonization in livestock^5^,^6^,^7^. Thus, *S. epidermidis* may benefit both humans and other animals and may help to control interspecies transmission of influenza viruses. (3) Many endogenous antiviral proteins, such as interferon (IFN), have been identified^21^, although they are normally activated many hours after infection. In contrast to these proteins, commensal bacteria in the human nasal cavity will be present the moment of infection to prevent influenza viral invasion.

Due to rapid antigenic drift, antigens for the development of flu vaccines must be updated annually based on global influenza surveillance. Antiviral drugs have limited efficacy if administered late in disease progression, and widespread use is likely to result in the emergence of resistant viral strains. Examination of how bacterial members of the nasal microbiome impact the infectivity of airborne pathogens may be key to controlling influenza infection. The use of commensal bacteria to inhibit pathogens may hold even greater potential than vaccines because these bacteria are natural competitors of pathogens, and their action does not require full host immune stimulation. We identified Embp, a fibronectin-binding protein that is shed from the bacterial surface, as an active and essential component of the *S. epidermidis* activity against influenza viruses. *S. epidermidis* bacteria are permanent inhabitants of the nasal cavity in humans, birds and pigs. Thus, the use of *S. epidermidis* and/or its derived products as anti-influenza agents may not only prevent viral invasion in humans but may also control the interspecies transmission of influenza viruses.

## Results

### Inhibition of influenza virus-induced erythrocyte haemagglutination by *S. epidermidis* culture supernatants

The inhibition of influenza virus-induced haemagglutination by *S. epidermidis* culture supernatants was measured using the haemagglutination inhibition (HI) test. Serial dilutions of *S. epidermidis* culture supernatants were reacted with 4 haemagglutination unit (HAU) of various influenza viruses followed by the addition of guinea pig erythrocytes. As shown in Table 1, the culture supernatants of both non-pathogenic strains (ATCC1457 and ATCC12228) of *S. epidermidis* demonstrated various degrees of inhibition of erythrocyte haemagglutination, indicating an antiviral activity of *S. epidermidis*. In contrast, the bacterial growth medium (TSB, tryptic soy broth) alone exhibited no inhibitory activity towards haemagglutination (data not shown), indicating that secreted factors of *S. epidermidis* in the culture supernatants, rather than nutrients in TSB, exerted the observed antiviral effect. In addition to two different clinically isolated strains of H1N1 influenza A virus, the bacterial supernatants also inhibited the erythrocyte haemagglutination caused by A/Aichi/2/68 (A/H3N2) and influenza B virus strain B/Brigit, suggesting that bacterial supernatants have broad-spectrum inhibitory activities against influenza viruses A and B.

Haemagglutination of erythrocytes is a common property of *S. epidermidis* strains^22^, which is related to adherence and biofilm formation and may be essential for the pathogenesis of biomaterial-associated infections caused by *S. epidermidis*. Interruption of the icaADBC operon essential for polysaccharide intercellular adhesin (PIA) synthesis by Tn917 insertions led to a haemagglutination-negative phenotype. Furthermore, the immunoglobulin G (IgG) fraction of antiserum to PIA greatly reduced erythrocyte haemagglutination. In addition, purified PIA led to a dramatic decrease in haemagglutination titres but did not mediate haemagglutination by itself. These results showed that PIA was the haemagglutinin of *S. epidermidis* or at least its major functional component. Recently, it was reported that *S. epidermidis* could produce poly-N-acetyl glucosamine cell surface polysaccharides to initiate erythrocyte haemagglutination^23^. To determine whether *S. epidermidis* secreted factors possess haemagglutination activities, which may compete with the ability of viruses to induce- haemagglutination, we incubated erythrocytes with the *S. epidermidis* (ATCC12228) culture supernatants alone in the absence of influenza virus. No haemagglutination activity was observed, indicating that the secreted factors of *S. epidermidis* themselves did not possess haemagglutination activities. These results are consistent with the fact that *S. epidermidis* (ATCC12228) is a PIA (icaADBC operon)-deficient strain^24^,^25^.

### A secreted factor of *S. epidermidis* reduces H1N1 virus-induced haemagglutination and is a papain-sensitive protein

A standard HI test was used to determine whether the secreted factors of *S. epidermidis* mediate the inhibition of virus-induced haemagglutination. We used 4 HAU of A/San Diego/1/09(H1N1 pdm09) as the haemagglutination antigen, and complete agglutination between the virus and the erythrocytes was observed. Consistent with the results shown in Table 1, *S. epidermidis* (ATCC12228) culture supernatants substantially reduced the erythrocyte haemagglutination induced by the influenza virus A/San Diego/1/09(H1N1 pdm09), with an HI titre of 1:16. However, following pretreatment by filtration or digestion with papain, the HI titre of bacterial supernatant was below the detection limit. These results suggested that the proteins that contributed to the antiviral activity of secreted bacterial factors were active components in the culture supernatants.

### Mass spectrometric identification of Embp as an active protein of *S. epidermidis* that inhibits virus-induced haemagglutination

**Figure 1:**
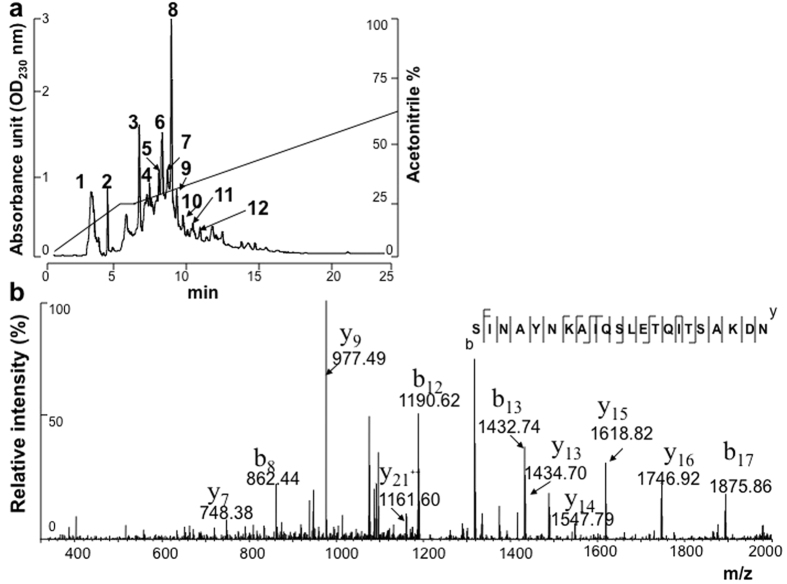
HPLC separation in combination with mass spectrometry for Embp identification. The proteins in the culture supernatants of *S. epidermidis* (ATCC12228) were separated by reversed-phase HPLC using a LUNA C18 5 μm column. (**a**) Twelve fractions were collected after HPLC separation. The HI activity of the eluted proteins (100 μl) in each fraction was tested using A/San Diego/1/09(H1N1 pdm09) as the virus antigen. (**b**) Tryptic digests of proteins in fraction 12 of the HLPC separation, which showed the greatest HI activity, were subjected to Nano-LC-LTQ MS/MS. A sequenced peptide (SINAYNKAIQSLETQITSAKDN) is shown and was identified as an internal peptide of Embp (Q8CP76). The m/z value of each “y” and “b” ion in collision-induced dissociation (CID) spectra is indicated.

To identify the *S. epidermidis* proteins that contribute to the antiviral effect, the culture supernatant of *S. epidermidis* (ATCC12228) was separated by reversed-phase high-performance liquid chromatography (HPLC) using a C18 column. Twelve major fractions (Fig. 1a) were obtained in 20 min of run time. Then, 100 μl of each fraction was collected for an HI test. The proteins eluted from fraction 12 exhibited the most potent reduction of A/H1N1(pdm09) virus-induced haemagglutination, and no inhibition of virus-induced haemagglutination was found in the other fractions (data not shown). The proteins in fraction 12 were digested with trypsin and then subjected to a nano liquid chromatography-linear ion trap quadrupole mass spectrometry (Nano-LC-LTQ MS/MS) analysis. One protein (Embp; 1 MDa; accession number: Q8CP76) was identified, and two internal peptides (SINAYNKAIQSLETQITSAKDN and QQVAEIIAQANKLNNEMGTLKT) of Embp were sequenced. The internal peptide (SINAYNKAIQSLETQITSAKDN; amino acid residues 3359–3380) of Embp is presented in Fig. 1b.

### Embp is essential for the antiviral activity of *S. epidermidis*

**Figure 2:**
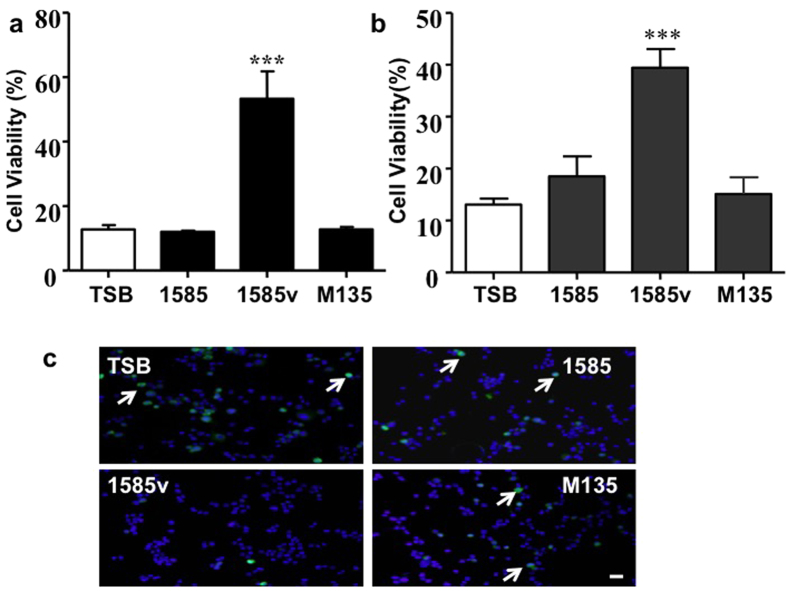
Mutation of Embp results in a loss of *S. epidermidis* antiviral activity against influenza virus. To assess the role of Embp in the inhibition of viral infectivity, the 0.22 μM filtered culture supernatants (100 μl) of wild-type or M135 (Embp mutant) *S. epidermidis* plus A/San Diego/1/09(H1N1 pdm09) (MOI = 1) were added to nasal (RPMI-2650) (**a**) or lung (A549) (**b**) epithelial cells (10^7^ cells) overni–ght. The incubation of cells with TSB medium plus the virus served as a control. Cell viability was determined using an ACP assay^48^. The wild-type [1585 (Embp negative), 1585 v (Embp positive)] and M135 (Embp mutant) strains were used in this experiment. The data represent the mean ± standard error (SE). ***P < 0.001 (one-way ANOVA followed by Dunnett’s multiple comparison test). (**c**) RPMI-2650 cells (10^7^ cells) were incubated with the NS1-GFP virus (MOI = 1) in the presence of TSB medium or culture supernatant of *S. epidermidis* 1585, 1585 v or M135 overnight in virus growth medium^47^. The replication of NS1-GFP-virus (arrows) and fluorescence were viewed with a Leica TCS SP2 confocal microscope. Bar = 25 μm.

An Embp mutant *S. epidermidis* strain was used to determine whether Embp is an essential protein in the *S. epidermidis*-mediated inhibition of influenza virus infection. To this end, we used the *S. epidermidis* strains 1585, 1585 v, and mutant M135^26^. *S. epidermidis* 1585 is a wild-type strain that produces no Embp after growth in TSB. In contrast, *S. epidermidis* 1585 v is a variant derived from strain 1585 in which, due to a chromosomal re-arrangement, a 460 kDa isoform of Embp is overexpressed even in TSB, while mutant M135 is an isogenic mutant of 1585 v in which expression of Embp is interrupted by insertion of transposon Tn917. The serially diluted culture supernatants (25 μl) of *S. epidermidis* 1585, 1585 v, and M135 were added to guinea pig erythrocytes in the presence of 4 HAU of A/San Diego/1/09(H1N1 pdm09) for the HI test as described in Table 1. The supernatant of *S. epidermidis* 1585 with no Embp expression did not affect virus-induced haemagglutination. In contrast, the supernatant of *S. epidermidis* 1585 v with Embp expression significantly blocked virus-induced haemagglutination, and this blockade of haemagglutination did not occur when the supernatant of *S. epidermidis* M135 was used (data not shown). To confirm this observation in human nasal cells, the culture supernatants (100 μl) of *S. epidermidis* 1585, 1585 v, and M135 were added to cultures of nasal RPMI-2650 (Fig. 2a) or lung (A549) (Fig. 2b) epithelial cells in the presence of A/San Diego/1/09(H1N1 pdm09). Consistent with results from the HI test, the supernatant of *S. epidermidis* 1585 v, but not 1585 or TSB medium alone, dramatically reduced the virus-induced cell death. However, the supernatant from *S. epidermidis* M135 completely blocked the bacterial inhibition of virus-induced cell death, suggesting that Embp is essential for the activity of *S. epidermidis* against viral infection.

Although HI tests indicated that the *S. epidermidis* culture supernatants possessed antiviral activity, they could not be used to measure viral colonization and invasion at the early stage of infection. Thus, nasal RPMI-2650 epithelial cells were infected with a recombinant influenza virus carrying a green fluorescent protein (GFP) reporter gene in the NS segment (NS1-GFP virus)^27^. To determine whether *S. epidermidis* could block the early entry of virus, RPMI-2650 cells were infected with the NS1-GFP virus in the presence of culture supernatants (100 μl) of *S. epidermidis* 1585, 1585 v, and M135. As shown in Fig. 2c, NS1-GFP viruses replicated in RPMI-2650 cells in the presence of TSB medium or culture supernatants of *S. epidermidis* 1585 or M135. However, this replication was dramatically impeded when cells were incubated with the supernatant of *S. epidermidis* 1585 v, suggesting that Embp plays a key role in limiting the early infection of the influenza virus in nasal cells.

### The interaction of *S. epidermidis* and the influenza virus

**Figure 3:**
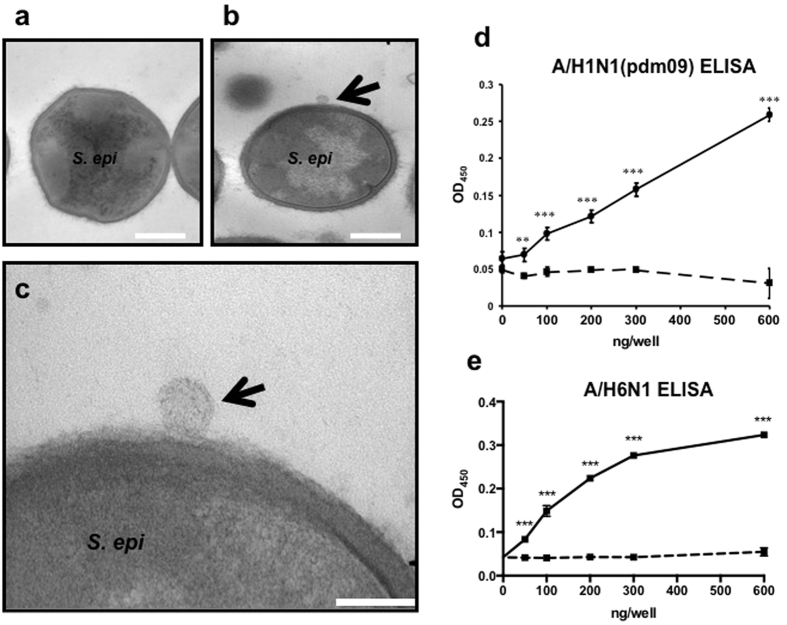
Influenza virus binds to *S. epidermidis* and rEmbp6599. *S. epidermidis* (ATCC1228; 10^6^ CFU) was incubated without (**a**) or with (**b,c**) A/San Diego/1/09(H1N1 pdm09) (32 HAU) in 1 ml virus growth medium for 24 h. Transmission electron microscopy analysis revealed that A/San Diego/1/09(H1N1 pdm09) (arrows) adhered to the surface of *S. epidermidis* [low (**b**) and high (**c**) magnification]. rEmbp6599 (a solid line) or BSA (a dashed line) was coated onto the 96-well plates (0–600 ng/well), and ELISA was used to determine the binding ability of rEmbp6599 to the influenza human isolate A/San Diego/1/09(H1N1 pdm09) (**d**) or the chicken isolate A/Chicken/Taiwan/2838/00(H6N1) (**e**). The data represent the mean ± standard error (SE) (n = 3, **P < 0.05; ***P < 0.001; by Student’s t-test, two-tailed, vs. BSA control). Bars (**a,b**) = 500 nm; Bar (**c**) = 150 nm.

To determine whether *S. epidermidis* could directly bind to influenza virus, *S. epidermidis* (ATCC12228) (10^6^ CFU) was incubated with A/San Diego/1/09(H1N1 pdm09) (32 HAU) in 1 ml virus growth medium for 24 h. Transmission electron microscopy demonstrated that the virus attached to the surface of *S. epidermidis* (Fig. 3a–c). To determine whether Embp could bind A/San Diego/1/09(H1N1 pdm09), a recombinant protein (rEmbp6599) containing an Embp domain (amino acids 6599–7340) was coated on enzyme-linked immunosorbent assay (ELISA) plates and reacted with A/San Diego/1/09(H1N1 pdm09). The rEmbp6599 protein contains seven found-in-various-architectures (FIVAR) (a fibronectin-binding domain) and eight protein G-related albumin-binding (GA) domains. As shown in Fig. 3d, the A/San Diego/1/09(H1N1 pdm09) virus adhered to rEmbp6599-coated ELISA plates in a dose-dependent manner. Similar results were obtained using avian influenza virus (AIV) H6N1 with the rEmbp6599-binding ELISA (Fig. 3e). Here, we illustrated that both *S. epidermidis* bacterial particles and Embp could directly bind to the virus. Embp without an LPXTG motif binds to the bacterial surface non-covalently, and this non-covalent attachment allows Embp to be released from the bacterial surface^26^. These results indicate that *S. epidermidis* may act as a natural guardian in the human nasal cavity, filtering influenza viruses from the nostrils by binding viruses via Embp.

### Embp decreases the mortality rate of influenza virus-infected chick embryos

**Figure 4:**
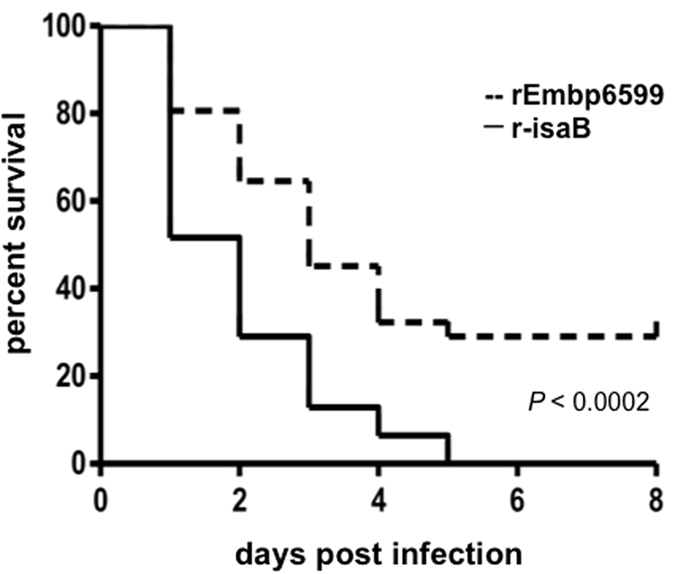
Embp increases the survival rate of embryonated eggs infected with influenza virus. Eggs were injected with 0.6 μg/100 μl rEmbp6599 (a solid line) or r-isaB (a dashed line) followed by infection with 50 EID_50_ of influenza virus A/Puerto Rico/8/34 (H1N1) on developmental day 11. Survival was monitored daily for 8 days, and the results are expressed as Kaplan-Meyer curves. The log rank test indicated that there were significant differences between eggs injected with viruses plus rEmbp6599 or r-isaB (*P* < 0.0002).

Embryonated eggs were used as an infection model of influenza to determine whether Embp could affect the mortality rate of virus-infected chick embryos. The 11-day-old embryonated eggs were injected with 100 μl rEmbp6599 (0.6 μg) followed by injection of 50 egg infective doses 50% (EID_50_) of influenza virus A/Puerto Rico/8/34 (H1N1). Eggs injected with the recombinant immunodominant surface antigen B (r-isaB) of *S. epidermidis* followed by injection of influenza virus were used as a control. Similar to Embp, isaB lacks an LPXTG motif and is both secreted and associated with the cell surface^28^. This protein is expressed in both *S. epidermidis* and *S. aureus*, and *S. epidermidis* isaB shares approximately 33% amino acid similarity with *S. aureus* isaB. As shown in Fig. 4, approximately 50% of the chick embryos injected with r-isaB and influenza virus died, whereas only 20% of the embryos injected with rEmbp6599 and influenza viruses died one day post-infection. All chick embryos injected with r-isaB and influenza virus died on day 5 post-infection, while approximately 30% of the chick embryos injected with rEmbp6599 and influenza virus survived to day 8 post-infection. These results indicated that injection of rEmbp6599 of *S. epidermidis* enhanced the survival rate of influenza virus-infected chick embryos.

### Embp reduced the tissue viral load and induced robust expression of antiviral cytokines in infected chickens

**Figure 5:**
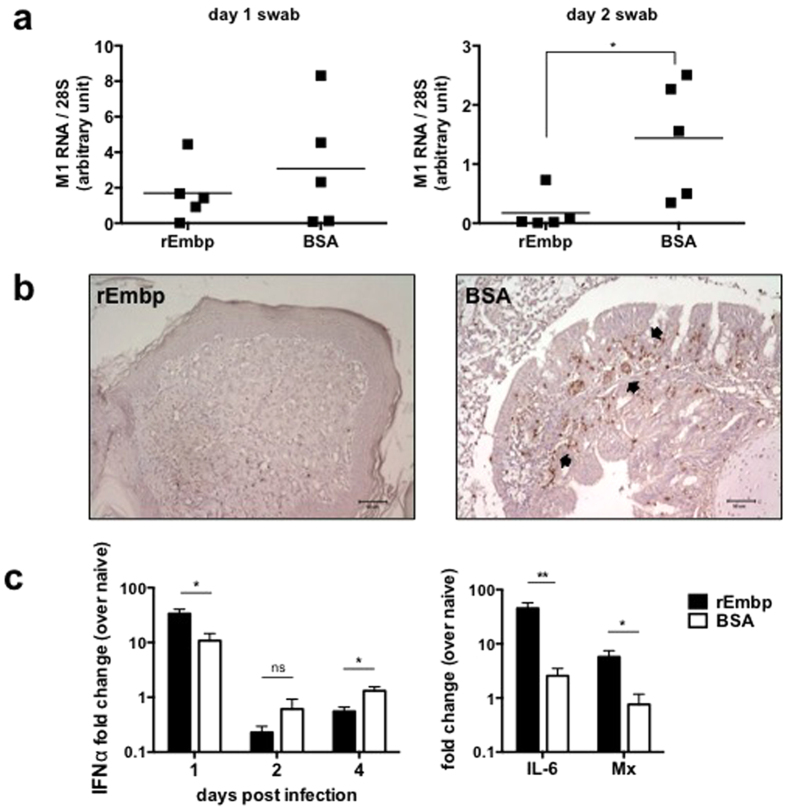
Embp reduces the tissue viral load and induces robust expression of antiviral cytokines in infected chickens. Chickens were intranasally administered 10 μg of rEmbp6599 or BSA as a control followed by intranasal challenge with 10^7^ EID_50_ of avian influenza A/Chicken/Taiwan/2838/00(H6N1) virus. (**a**) Chicken throat swabs were sampled for viral load analysis. The mRNA level of the viral M1 gene relative to cellular 28S rRNA was determined using real-time RT-PCR. The results are expressed in arbitrary units. (**b**) Chicken nasal turbinates from rEmbp-inoculated or BSA-inoculated chickens were harvested after sacrifice on day 4 post-infection. Immunohistochemistry was performed using virus-specific chicken antiserum. Arrows indicate the positive AIV antigen signals. Images are representative of two independent experiments. (**c**) Levels of IFN-α, IL-6, and Mx mRNA in the cells sampled from the throat tissues were determined by qRT-PCR and normalized to 28S rRNA. Relative gene induction is expressed as a fold-change over naive (uninfected) chickens. Each time point represents five chickens, with the mean ± SEM from the replicates. Real-time PCR experiments were performed in triplicate. The data between the two groups of chickens were compared by two-tailed Student’s t-test. *P < 0.05, **P < 0.01.

A chicken infection model was employed to investigate the *in vivo* antiviral efficacy of Embp. Prior to AIV H6N1 challenge, a group of chickens received 10 μg of rEmbp6599, while another group received bovine serum albumin (BSA) as a control. Throat swabs were taken daily during the experiment for viral load assessment. As shown in Fig. 5a, a lower viral load was detected in the rEmbp-treated chickens at days 1 and 2 post-infection compared with the BSA-treated chickens. In addition, after euthanasia on day 4 post-infection, the tracheas were harvested for viral detection. The results showed that 100% (5/5) of the tracheas from BSA-treated chickens tested positive, whereas only 20% (1/5) of the tracheas from rEmbp-treated chickens tested positive for the virus (data not shown). Furthermore, the presence of AIV antigens in nasal turbinates was investigated using immunohistochemistry. As indicated in Fig. 5b, fewer AIV antigens were detected in chickens that received rEmbp (left) compared with control chickens (right).

To further examine the expression profile of cytokines, we investigated the local α-interferon (IFN-α), interleukin (IL)-6, and Mx response in the cells sampled from the throat tissues by qRT-PCR and obtained the relative mRNA expression levels in virus-challenged chickens compared to naïve (uninfected) chickens. On day 1 post-infection, the rEmbp group showed higher levels of IFN-α mRNA compared to the BSA control group (33.54-fold vs. 10.79-fold, respectively) (Fig. 5c). Similarly, the levels of IL-6 and Mx mRNA from the rEmbp group were higher than those from the BSA control group on day 1 post-infection. The presence of higher levels of antiviral cytokines collectively supports our finding of a lower viral load in rEmbp-treated chicken tissues. However, the IFN-α expression of BSA control chickens was found to be higher than that of the rEmbp group during days 2–4 post-infection, which may have been induced by the greater amount of residual virus in the tissues. In summary, our study shows that intranasal application of rEmbp can protect chickens from infection with low pathogenic AIV, which replicates mainly in the upper respiratory tract.

## Discussion

At least two possibilities may explain human susceptibility to influenza virus infection in the presence of commensal *S. epidermidis* in the nasal cavity. First, the amount of *S. epidermidis* and/or Embp in the nasal cavity may be too low to confer full protection. Second, pre-existing naturally occurring antibody (NAb) to Embp may be produced in hosts, thereby neutralizing the antiviral activity of Embp. Indeed, because NAb (IgG^29^ and IgA^30^) to *S. epidermidis* has been detected in humans, it would be worthwhile to measure the titre and neutralizing activity of NAb to Embp. *S. epidermidis* is the dominant commensal bacterium, accounting for approximately 10% of all bacteria found in the human nasal cavity^1^. A recent publication indicated that *S. epidermidis* colonizes the nasal cavity of 96% (58/60) of healthy people^12^. Another study found that *S. epidermidis* had a 100% colonization rate in the nares of 156 healthy volunteers^31^. The Embp (Q8CP76) of *S. epidermidis* (ATCC12228) shares approximately 32% identity with Embp (Q931R6) of *S. aureus* (Mu50), although the anti-influenza virus activity of *S. aureus* Embp has yet to be determined.

The full-length Embp (1 MDa) harbours FIVAR and GA domains^26^,^32^. The rEmbp2588 (amino acids 2588–3187) solely containing FIVAR domains exhibited no biofilm-inducing activity. In contrast, rEmbp6599 (amino acids 6599–7340) containing both the FIVAR and GA domains induced biofilm formation^33^. Previously, it was shown that the 32 kDa recombinant Embp32 protein with FIVAR/GA domains could prevent *S. epidermidis*, but not *S. aureus*, from binding to fibronectin^32^. Our results demonstrated that the culture supernatant of *S. epidermidis* 1585 v, which contained the N-terminal half of truncated Embp, significantly reduced viral infectivity (Fig. 2). Thus, we hypothesize that the C-terminal half, which includes FIVAR, GA and DUF1542 (amino acids 9445–9831), is responsible for blocking influenza virus infection. The rEmbp2588 protein has the capability to bind fibronectin without inducing biofilm formation^26^. If rEmbp2588 possesses anti-influenza virus activity, the possibility of biofilm formation induced by Embp will be avoided when Embp is used as an antiviral agent. Embp binds to fibronectin type III 12–14 domains, with fibronectin domain III12 suggested as a binding site^26^. The alpha5 beta1 integrin primarily binds to the RGD-containing domain (Type III 7–10) of fibronectin^34^. Thus, Embp and integrin bind different domains of fibronectin. Fibronectin is a glycoprotein and contains both α2-3 and α2-6-linked sialic acids. Previous findings demonstrated that fibronectin directly interacts with the envelope glycoproteins of the influenza virus^35^,^36^. However, this binding was abrogated when fibronectin was pre-treated with neuraminidase, suggesting that the sialic acids of fibronectin are responsible for the affinity to the virus.

Although it is unclear how Embp binds to viruses, binding may be mediated by fibronectin. Embp does not possess a LPXTG motif^26^, suggesting a non-covalent attachment to the cell surface, which also explains why this protein was identified in bacteria-free supernatants. In our laboratory, influenza viruses are isolated from Madin-Darby canine kidney (MDCK) cells and stored in the cell culture supernatants. The cell supernatants contain cell medium and foetal bovine serum, which is a source of exogenous fibronectin. As shown in Fig. 3, fibronectin in the viral stocks may act as a bridge, connecting *S. epidermidis* Embp to the viruses. However, cell medium containing exogenous fibronectin did not inhibit virus-induced haemagglutination and viral colonization, suggesting that fibronectin alone is not sufficient for inhibiting the infectivity of the influenza virus. Our results demonstrate that the anti-influenza activity of *S. epidermidis* was markedly restrained when Embp was mutated, confirming that Embp is indispensable for *S. epidermidis* function against viral infection (Fig. 2). Our findings also indicate that both *S. epidermidis* bacterial particles and Embp can directly bind to two different influenza viruses (Fig. 3). The non-covalent attachment allows Embp to be released/shed from bacterial surfaces. Based on these results, we suggest that *S. epidermidis* in the host nasal cavity acts as a natural particle, filtering influenza viruses from host nostrils through the binding of virus to Embp, which is located on bacterial surfaces and released into the extracellular milieu. Thus, *S. epidermidis* in the nasal microbiome may function as a front-line defender against the early steps of influenza virus infection.

Although it remains to be determined whether Embp can bind to influenza virus in the absence of fibronectin, the Embp domains responsible for viral binding (neutralization) and infectivity reduction (viral internalization) may be different. The viral neutralization of rEmbp6599 alone may be insufficient to confer complete protective immunity *in ovo* (Fig. 4) and *in vivo* (Fig. 5). Thus, full protection may require a full-length or larger recombinant Embp that can both bind viruses and reduce infectivity. Fibronectin is known to be present in the chick embryo^37^. However, chicken and mammal fibronectins are differentially glycosylated^38^. If fibronectin contributes extensively to the anti-influenza activity of Embp, the distinction of fibronectins in chickens and humans may provide an explanation for the partial protection of Embp-injected chick embryos from virus infection. Augmentation therapy using purified α1-antitrypsin (A1AT), such as human plasma-pooled Prolastin^®^, has been used clinically for the treatment of chronic obstructive pulmonary disease (COPD)^39^. The use of recombinant Embp as a preventive or therapeutic antiviral agent may have its own set of challenges because (1) Embp may be immunogenic and (2) Embp proteins may not remain stable over a long period of time in the nasal passage. Enhancing the stability or decreasing the immunogenicity of Embp by PEGylation^40^,^41^ may be needed before Embp can be used as clinical augmentation therapy for limiting influenza virus infection.

Here, we demonstrate that Embp of *S. epidermidis*, a nasal commensal bacterium, exhibits *in vitro*, *in ovo*, and *in vivo* anti-influenza virus activity. Unlike antibiotics, products from commensal bacteria are indigenous molecules in humans, which may have a lower risk of causing serious complications or generating virus resistance. The significance of this study includes (1) noting that inappropriate use of antibiotics may eliminate human commensals, making it more difficult to fight influenza; (2) validating the function of human commensals as endogenous viral inhibitors, which suggests that new research on the nasal microbiome^1^,^42^ against influenza virus infection should be undertaken. Indeed, antiviral agents targeting an initial site (nasal cavity) of infection may be effective at preventing the systemic spread of viral infections; (3) the use of Embp as augmentation therapy may complement the inability of current antiviral drugs to fight unknown viral strains; and (4) *S. epidermidis* bacteria are also commensals in the nasal cavities of birds^5^,^6^,^7^,^18^,^19^ and pigs^19^,^20^. Thus, these bacteria and/or their derived products may be used as interfering agents^5^,^6^,^7^ in animal farms (e.g., chicken fields) using aerosol sprays in large-scale operations, and this approach may efficiently control the interspecies transmission of the influenza virus from animals to humans.

## Methods

### Ethics statement

Experiments involving embryonated eggs were performed at the University of California, San Diego (UCSD). The UCSD ethics committee specifically approved this study under an approved Institutional Animal Care and Use Committee (IACUC) protocol (no. S10058). The experimental chicken study was conducted at National Taiwan University, under an approved IACUC protocol (no. NTU-103-EL-3). All animal experiments were carried out in accordance with the approved guidelines.

### Virus growth

Two clinically isolated influenza H1N1 viruses [seasonal A/San Diego/51/08 and A/San Diego/1/09(H1N1 pdm09)], the NS1-GFP virus and various subtypes of influenza virus obtained from ATCC were grown in virus growth medium containing Dulbecco’s modified Eagle’s medium (DMEM) with glutamine, 0.2% BSA, 25 mM HEPES buffer and 2 μg/ml TPCK-trypsin (Sigma, St. Louis, MO) and propagated in monolayers of MDCK cells^43^. The avian influenza virus strain A/Chicken/Taiwan/2838/00(H6N1) was isolated from a chicken flock in Taiwan, and the virus was propagated in 10-day-old specific pathogen-free (SPF) chicken embryos^44^.

### Haemagglutination (HA) test and HI test

HA and HI tests were performed in v-bottom 96-well microtiter plates (Costar, Cambridge, MA) at room temperature using 0.5% guinea pig erythrocytes (Lonza, Walkersville, Inc., MD), following previously described protocols^45^,^46^. For the HI test, in brief, pre-determined 4 HAU influenza virus was mixed with 25 μl of each dilution of culture supernatants (10 μg/ml) of *S. epidermidis* at room temperature for 40 min. The HI titre is the highest dilution of culture supernatant that causes complete inhibition of 4 HAU of antigen. In one experiment, the culture supernatants of *S. epidermidis* were filtered with a MacroSep 10 kDa OMEGA cutoff column (Pall Gelman, Dreieich, Germany) or digested with papain (10 mg/ml) for 4 h before adding them to the mixture of erythrocytes and virus. The data presented are representative of three separate experiments with similar results.

### Protein identification via HPLC in conjunction with Nano-LC-LTQ MS/MS analysis

The proteins in the culture supernatants of *S. epidermidis* (ATCC12228) were separated by reversed-phase HPLC using a LUNA C18 5 μm column, 250 × 4.6 mm (Phenomenex, Torrance, CA). The gradient mobile phase consists of buffer A [HPLC grade water plus 0.1% trifluoroacetic acid (TFA)] and buffer B (acetonitrile plus 0.1% TFA). The flow rate was set to 1.0 ml/min, and proteins were detected at 230 nm. Twelve fractions were collected after HPLC separation. The antiviral activity of the eluted proteins (100 μl) in each fraction was tested with an HI test using A/San Diego/1/09(H1N1 pdm09). The eluted proteins in fraction 12 with the most potent inhibition of virus-induced haemagglutination were digested with trypsin and subjected to Nano-LC-LTQ MS/MS analysis (Thermo Scientific, San Jose, CA)^47^. The automated Nano-LC-LTQ MS/MS setup consisted of an Eksigent Nano 2D LC system, a switch valve, a C18 trap column (Agilent, Santa Clara, CA), and a capillary reversed-phase column (10 cm in length, 75 mm internal diameter) packed with 5 mm, C18 AQUASIL resin with an integral spray tip (Picofrit, 15 mm tip, New Objective, Woburn, MA). A reversed-phase LC directly coupled to a LTQ ion trap mass spectrometer (Thermo Scientific, San Jose, CA) was run using a linear gradient elution from buffer A (H_2_O plus 0.1% formic acid) to 50% buffer A plus 50% buffer B (acetonitrile plus 0.1% formic acid) in 100 min. The instruments were operated in the data-dependent mode. The data on the four strongest ions above an intensity of 26,105 were collected with dynamic exclusion enabled and the collision energy set at 35%. Large-scale MS/MS spectra were extracted using the default value by BioworksH 3.2. Charge state deconvolution and deisotoping were not performed. All MS/MS spectra were analysed using the in-house Sorcerer^TM^ 2 system with SEQUEST (v.27, rev. 11) as the search programme for protein identification. SEQUEST was set up to search the target-decoy ipi.MOUSE.v3.14 database containing protein sequences in both forward and reverse orientations (68,627 entries in each orientation) using trypsin as the digestion enzyme with an allowance of up to five missed cleavages. The false positive rates were roughly determined by doubling the ratio between the number of decoy hits and the total number of hits. SEQUEST was searched with a fragment ion mass tolerance of 0.5 Da and a parent ion tolerance of 1.0 Da.

### Cell viability and virus entry

Human nasal epithelial cells (RPMI-2650; ATCC CCL-30) were cultured in Eagle’s minimum essential medium (MEM) supplemented with 10% foetal calf serum (FCS) at 37 °C and 5% CO_2_. Human lung epithelial cells (A549; ATCC CCL-185) were grown in Roswell Park Memorial Institute (RPMI) media containing 10% foetal bovine serum (FBS) at 37 °C and 5% CO_2_. Cells (10^7^) were incubated with the 0.22 μM filtered culture supernatants (100 μl) of wild-type or M135 *S. epidermidis* in the presence of A/San Diego/1/09(H1N1 pdm09) [multiplicity of infection (MOI) = 1] or TSB medium overnight. Cell viability was quantified with an acid phosphatase (ACP) assay^48^. Virus entry was determined by incubation of RPMI-2650 cells (10^7^ cells) with the NS1-GFP virus (MOI = 1) in the presence of TSB medium or culture supernatant of *S. epidermidis* 1585, 1585 v or M135^26^ overnight in virus growth medium containing 2 μg/ml TPCK-trypsin at 37 °C and 5% CO_2_. The GFP fluorescence in cells counter-stained with the blue fluorescent Hoechst 33342 nuclear dye was viewed with a Leica TCS SP2 confocal microscope (Leica Microsystems, Inc., Buffalo Grove, IL).

### Expression of r-Embp and r-isaB

The construction of *Escherichia coli* (*E. coli*) BL21AI overexpressing Embp6599 (amino acids 6599–7340) using a pDEST17 expression vector (Invitrogen, Karlsruhe, Germany) has been previously described^26^. The expression of Embp6599 was induced by 0.2% L-arabinose for 4 h. Then, r-Embp6599 was affinity-purified using a HiTrap chelating HP column (GE Healthcare Biosciences, Pittsburgh, PA). Purified r-Embp6599 was detected by 12.5% sodium dodecyl sulphate polyacrylamide gel electrophoresis (SDS-PAGE) stained with Coomassie brilliant blue (see Supplementary Fig. S1a). A vector encoding isaB (gi|57636443) was constructed by inserting the isaB PCR product into the p*Ecoli*-Nterm 6xHN vector (Clontech Laboratories, Inc., Mountain View, CA) at the SaII and HindIII restriction sites. Specific primers, including the sense (5′-AATAGTCGACATGAAAAGGTTTGCAAAAGCATTTG-3′) and anti-sense primers (5′-CAGAATTCTTATGACAATGTAGCACTTGTGACATACC-3′), were designed to clone isaB from *S. epidermidis* (ATCC12228). The expression of isaB from the *E. coli* vectors was detected in the absence or presence of 1 mM isopropyl-β-D-thiogalactoside (IPTG) (see Supplementary Fig. S1b). The r-isaB was obtained via an In-Fusion Ready TALON Express Bacterial Expression and Purification kit (Clontech Laboratories, Inc., Mountain View, CA) and purified via a standard nickel resin purification protocol (Qiagen, Valencia, CA).

### Transmission electron microscopy

*S. epidermidis* (ATCC12228) (10^6^ CFU) was incubated with A/San Diego/1/09(H1N1 pdm09) (32 HAU) in 1 ml virus growth medium for 24 h. After centrifugation of the mixture of bacteria and viruses at 5,000× *g* for 10 min, the pellets were fixed in Karnovsky’s fixative (4% paraformaldehyde, 2.5% glutaraldehyde, 5 mM CaCl_2_ in 0.1 M Na cacodylate buffer, pH 7.4) overnight at 4 °C, followed by 1% OsO_4_ in 0.1 M Na cacodylate buffer, pH 7.4, *en bloc* staining with 4% uranyl acetate in 50% ethanol, and subsequent dehydration using a graded series of ethanol solutions followed by propylene oxide and infiltration with epoxy resin (Scipoxy 812, Energy Beam Sciences, Agawam, MA). After polymerization at 65 °C overnight, thin sections were cut and stained with uranyl acetate (4% uranyl acetate in 50% ethanol) followed by bismuth subnitrate. Sections were observed at an accelerating voltage of 60 kV with a Zeiss EM10C electron microscope (Carl Zeiss, Thornwood, NY).

### ELISA

To investigate the binding of rEmbp6599 to the influenza virus, 96-well ELISA polystyrene plates (Corning Life Sciences, Lowell, MA) were used. The rEmbp6599 or BSA (0-600 ng/well) was coated on ELISA plates and incubated with a 1/200 dilution of A/San Diego/1/09(H1N1 pdm09) (32 HAU) for 2 h followed by addition of a polyclonal antibody (IgG; 1:1,000) to A/San Diego/1/09(H1N1 pdm09) for 1 h. The polyclonal antibody was generated in rabbits by immunization with the human influenza A (H1N1) 2009 monovalent vaccine (CSL Biotherapies Inc., King of Prussia, PA). Secondary horseradish peroxidase (HRP)-labelled donkey anti-rabbit antibodies were then added. For binding assays against H6N1 AIV, A/Chicken/Taiwan/2838/00 was used to react with the coating antigen (rEmbp or BSA). After washing, chicken antiserum against A/Chicken/Taiwan/2838/00 at a dilution of 1:2,000 was added to the wells. After further washes, HRP-conjugated rabbit anti-chicken IgY (KPL, Gaithersburg, MD) at a 1:2,000 dilution was incubated for an additional hour at room temperature. The 3,3′,5,5′-tetramethylbenzidine (TMB) substrate (Bio-Rad Labs, Hercules, CA) was then added, and the reaction was stopped using 1 N sulfuric acid (H_2_SO_4_). The optical density (OD) was measured on an ELISA plate reader at 450 nm.

### Influenza virus infections in embryonated eggs

Ten-day-old SPF chicken embryonated eggs were obtained from a local producer (McIntyre Poultry & Fertile Eggs, Lakeside, CA). Eggs were incubated at 37 °C and 50 to 60% relative humidity in a specialized incubator (RX1, Lyon Technologies, Chula Vista, CA) for one day before infection with the influenza virus. After the shells of 11-day-old embryonated eggs were wiped with a povidone-iodine prep pad (Medline Industries, Inc., Mundelein, IL), they were perforated at the blunt end and on the side using a hand drill. After perforation of the shell membrane, eggs were injected with 100 μl rEmbp6599 or r-isaB (0.6 μg) followed by injection of 50 EID_50_ of influenza virus [A/Puerto Rico/8/34 (H1N1) (ATCC)] (100 μl) via the allantoic route using a sterile 1-ml syringe. The holes were then sealed using an ethylene vinyl acetate glue gun. Survival was monitored for up to 8 days by daily candling. The EID_50_ was calculated using a previously published method^49^. Thirty-one eggs per group were used for three independent experiments. Survival data were plotted as Kaplan-Meyer curves and were analysed statistically by a log-rank test using GraphPad Prism version 5.00 for Windows (GraphPad Software, San Diego, CA).

### Influenza virus infections in SPF chickens

Ten four-week-old SPF chickens were obtained from the Animal Health Research Institute, Taiwan and were randomly divided into two groups. Chickens were inoculated with 10 μg of rEMbp6599 or BSA as a control, followed by intranasal challenge with the avian influenza virus A/Chicken/Taiwan/2838/00(H6N1)^44^ (10^7^ EID_50_ per chicken). During the experiment, throat swabs were sampled daily for examination of viral titre and cytokine production. All chickens were euthanized at day 4 post-infection, and nasal turbinates and tracheas were harvested for further investigation.

### Virus detection in chicken throat swabs and tracheas

RNA in chicken throat swabs and tracheas was extracted using TRIzol reagent (Invitrogen) according to the manufacturer’s instructions. To measure the viral load in the throat swabs, real-time RT-PCR was performed with iScript (Bio-Rad) and an iQ SYBR Green Supermix Kit (Bio-Rad) using previously described primers that target the M1 gene of the influenza A virus^50^ and chicken 28S^51^. Real-time PCR experiments were performed in triplicate. The data are expressed in arbitrary units. For viral detection in the trachea tissues, a standard one-step RT-PCR was performed with primers (NP1200f/NP1529r) that target the nucleoprotein gene of the influenza virus^52^.

### Cytokine expression analysis

The production of antiviral cytokines was evaluated from the cells sampled from the throat tissues. Chicken IFN-α, IL-6, and Mx1 expression levels were analysed by real-time RT-PCR using previously described primers^51^,^53^. All reactions were set up in triplicate, and the obtained cycle threshold (Ct) values were normalized to 28S. The relative expression of cytokines (fold change of uninfected control) was determined using the 2^−ΔΔCt^ method.

### Immunohistochemistry

Nasal turbinates collected from chickens were immediately fixed in 10% buffered formalin, followed by dehydration in absolute ethanol, and paraffin wax embedding. Tissue section slides were prepared as previously described^54^. Sections were blocked by 5% normal goat serum (Invitrogen) in PBS for 10 min and stained with chicken antiserum against A/Chicken/Taiwan/2838/00(H6N1) at a 1:500 dilution for 4 h at room temperature. After washing, the sections were stained with HRP-conjugated rabbit anti-chicken IgY (KPL) at a 1:500 dilution for 1 h at room temperature. The signal was visualized using 3,3′-diaminobenzidine (DAB; Thermo Fisher Scientific), and the sections were counterstained with Mayer’s haematoxylin.

## Additional Information

**How to cite this article**: Chen, H.-W. *et al*. Nasal commensal *Staphylococcus epidermidis* counteracts influenza virus. *Sci. Rep.* **6**, 27870; doi: 10.1038/srep27870 (2016).

## Supplementary Material

Supplementary Information

## Figures and Tables

**Table 1:** The HI titres of *S. epidermidis* culture supernatants against various influenza virus strains.

Culture sup	A/San Diego/51/08(H1N1)		A/San Diego/1/09(H1N1 pdm09)		A/Aichi/2/68(H3N2)		B/Brigit	
	ATCC1457	ATCC12228	ATCC1457	ATCC12228	ATCC1457	ATCC12228	ATCC1457	ATCC12228
HI titre	NT^a^	1:16	NT^a^	1:16	1:4	1:4	1:8	1:8

^a^NT: not tested.
