# Supplementary material for: Nasal commensal Staphylococcus epidermidis counteracts influenza virus
Source: Sci Rep. 2016 Jun 16;6:27870. doi: 10.1038/srep27870 (PMC4910069; doi:10.1038/srep27870)

Nasal commensal *Staphylococcus epidermidis* counteracts influenza virus

Hui-Wen Chen[1]*, Pei-Feng Liu[2], Yu-Tsueng Liu[3,5], Sherwin Kuo[2], Xing-Quan Zhang[3], Robert T. Schooley[3], Holger Rohde[4], Richard L. Gallo[2], and Chun-Ming Huang[2,5] *

[1]Department of Veterinary Medicine, National Taiwan University, Taipei, Taiwan
[2]Department of Dermatology, University of California, San Diego, CA, USA
[3]Division of Infectious Diseases, Department of Medicine, University of California, San Diego, CA, USA
[4]Institut für Medizinische Mikrobiologie, Virologie und Hygiene, Universitätsklinikum, Hamburg, Germany
[5]Moores Cancer Center, University of California, San Diego, CA, USA

Supplementary Figure S1. The expression of r-Embp and r-isaB. The construction of *E. coli* over-expressing *S. epidermidis* Embp6599 or isaB was described in Methods. The expression of r-Embp6599 (A) or r-isaB (B) from the *E. coli* was detected in the absence (lane 1) or presence (lane 2) of 0.2% L-arabinose or 1 mM IPTG, respectively. Purified rEmbp6599 or r-isaB (Lane 3) was obtained via a HiTrap chelating HP column or In-Fusion Ready TALON Express Bacterial Expression and Purification kit, respectively.

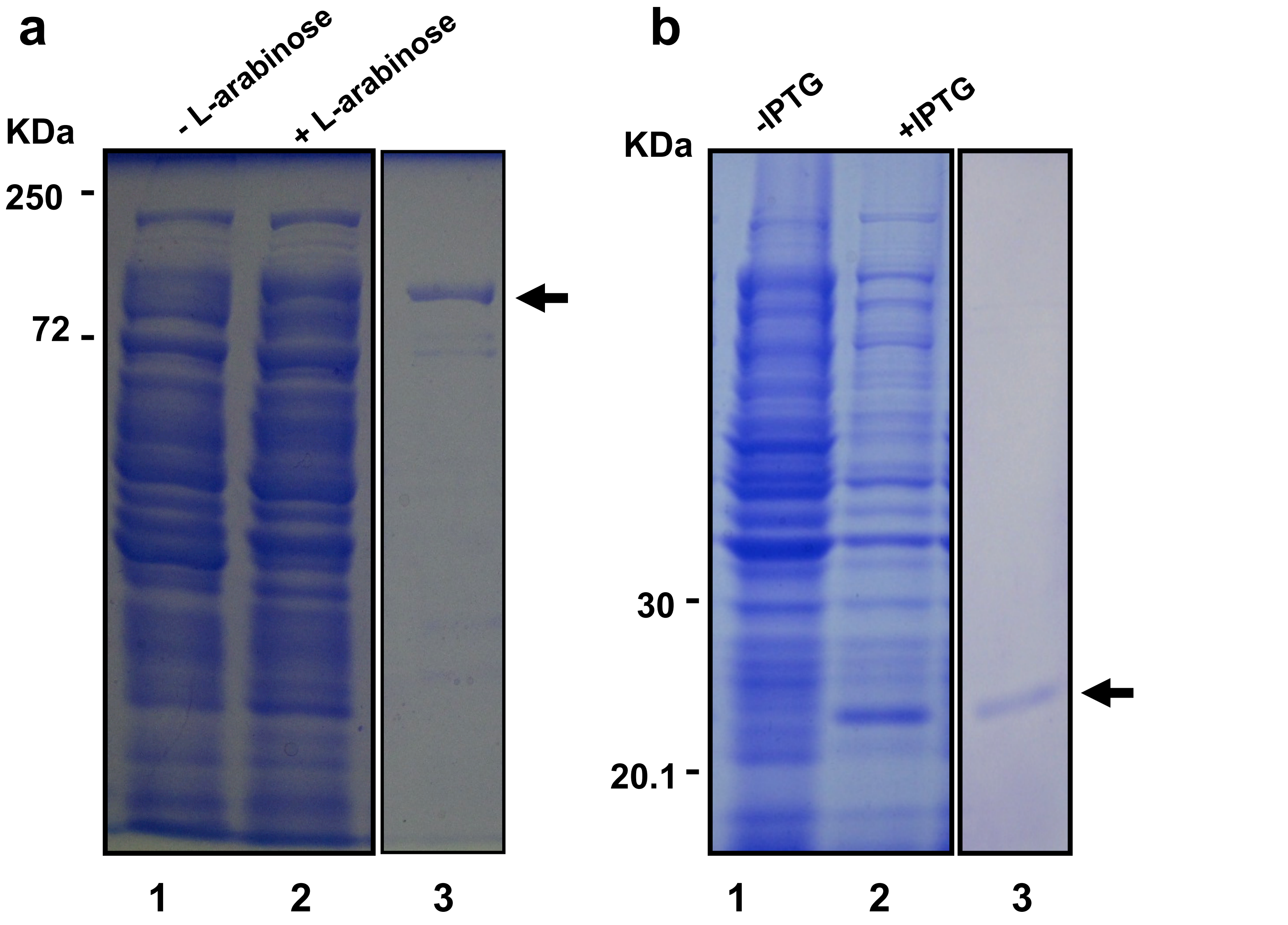

Supplement: Supplementary Information [file srep27870-s1.pdf]
